# Opposing effects of antibiotics and germ-free status on neuropeptide systems involved in social behaviour and pain regulation

**DOI:** 10.1186/s12868-020-00583-3

**Published:** 2020-07-22

**Authors:** Katerina V. A. Johnson, Philip W. J. Burnet

**Affiliations:** 1 Department of Experimental Psychology, University of Oxford, Radcliffe Observatory Quarter, Oxford, OX2 6GG UK; 2 Department of Psychiatry, Warneford Hospital, University of Oxford, Oxford, OX3 7JX UK

**Keywords:** Microbiome, Germ-free, Antibiotics, Μ-opioid, Oxytocin, Vasopressin, Neuroreceptors, Neuropeptides

## Abstract

**Background:**

Recent research has revealed that the community of microorganisms inhabiting the gut affects brain development, function and behaviour. In particular, disruption of the gut microbiome during critical developmental windows can have lasting effects on host physiology. Both antibiotic exposure and germ-free conditions impact the central nervous system and can alter multiple aspects of behaviour. Social impairments are typically displayed by antibiotic-treated and germ-free animals, yet there is a lack of understanding of the underlying neurobiological changes. Since the μ-opioid, oxytocin and vasopressin systems are key modulators of mammalian social behaviour, here we investigate the effect of experimentally manipulating the gut microbiome on the expression of these pathways.

**Results:**

We show that social neuropeptide signalling is disrupted in germ-free and antibiotic-treated mice, which may contribute to the behavioural deficits observed in these animal models. The most notable finding is the reduction in neuroreceptor gene expression in the frontal cortex of mice administered an antibiotic cocktail post-weaning. Additionally, the changes observed in germ-free mice were generally in the opposite direction to the antibiotic-treated mice.

**Conclusions:**

Antibiotic treatment when young can impact brain signalling pathways underpinning social behaviour and pain regulation. Since antibiotic administration is common in childhood and adolescence, our findings highlight the potential adverse effects that antibiotic exposure during these key neurodevelopmental periods may have on the human brain, including the possible increased risk of neuropsychiatric conditions later in life. In addition, since antibiotics are often considered a more amenable alternative to germ-free conditions, our contrasting results for these two treatments suggest that they should be viewed as distinct models.

## Background

The regulation of behaviour and emotion is complex, influenced by both genes and the environment. The microbial environment within the gut, known as the gut microbiome, has recently been shown to affect various aspects of brain development and behaviour [1–3]. The brain is particularly sensitive to perturbations throughout childhood and adolescence when its structure is undergoing rapid change [4, 5]. During this time, environmental disruptions may permanently impact brain function and increase susceptibility to neuropsychiatric conditions. These include changes to the gut microbiome which can affect neurodevelopment via gut–brain signalling. The microbial community of the gut may influence the functioning of the central nervous system through various mechanisms, including communication via the nervous, immune and endocrine systems [6–8].

Numerous studies in rodents have revealed that manipulation of the gut microbiome can affect the brain’s anatomy and physiology, as well as behaviour [1–3]. In particular, multiple facets of social behaviour are influenced by the gut microbial community [9, 10]. Germ-free animals provide a useful model to directly investigate which aspects of neurodevelopment and behaviour are modulated by the gut microbiome since they are raised in a sterile environment with no associated microorganisms. The germ-free phenotype exhibits behavioural traits reflective of autism, such as a reduced preference for social interactions [11–15] and social novelty [12, 13, 16], as well as repetitive behaviours [12]. However, if germ-free mice are colonized at weaning with gut microbiota from conventionally raised mice, their deficits in social interaction and repetitive behaviours can be reversed [12]. While the majority of studies using the standard three-chamber social interaction test [17] have found that germ-free mice are less sociable [12–15], one study has reported an increase in sociability [18]. This may be due to different experimental conditions such as the age of the mice being tested, the strain of stimulus mouse used in the social interaction test and animal husbandry practices. Nonetheless, germ-free studies reveal that the gut microbial community is important for normal social development.

In addition, recent research provides strong support for the causal relationship between a dysbiotic gut microbiome and altered social behaviour [13]. Offspring of mice which had been fed a high-fat diet exhibited autistic-like behaviours including social impairments, anxiety and repetitive behaviours, as well as fewer oxytocin-expressing neurones in the hypothalamus. Notably, offspring were found to have an altered gut microbial community but supplementation with the bacterial species *Lactobacillus reuteri* restored oxytocin levels and reversed social deficits in this mouse model of autism.

Another commonly used approach to investigate the effect of the gut microbiome on the brain and behaviour is antibiotic treatment to deplete the gut microbial community during specific developmental periods [19]. Antibiotics also alter the composition and function of the gut microbiome, as well as reducing its diversity [20]. Studies in rodents have demonstrated that antibiotic treatment can cause visceral pain [19, 21, 22], cognitive deficits [19, 23, 24] and behavioural changes, including impairments in social behaviour [19, 23, 25–29]. For example, early-life antibiotic exposure to low-dose penicillin can have lasting effects on both gut microbiome composition and behaviour, with mice showing decreased sociability and a reduced preference for social novelty [27, 29]. However in both studies, supplementation with *Lactobacillus rhamnosus* at the same time as antibiotic treatment protected animals from these social impairments. Similarly, mice administered an antibiotic cocktail during the key developmental stage of early adolescence have an altered microbiome composition and exhibit cognitive and social deficits [23]. They also show reduced expression of the neuropeptides vasopressin and oxytocin, with the latter only reduced when the animals have been exposed to stress [23]. In addition, administration of *L*. *reuteri* in mice has been found to increase plasma oxytocin through vagal signalling [30].

While studies have frequently reported the impact of an altered gut microbiome on host social behaviour, there is little understanding of the underlying neurochemical changes. We therefore aimed to address this by investigating the effect of antibiotic treatment and germ-free status on the gene expression of neuropeptides and their receptors implicated in social behaviour. Mammalian social behaviour is underpinned by multiple neuropeptide signalling pathways, namely the μ-opioid, oxytocin and vasopressin systems [31, 32], which have evolutionarily conserved functions in regulating social behaviour [33]. Specifically, both oxytocin and vasopressin are well known for their roles in social cognition, pair bonding and sociosexual behaviours [33, 34]. The endogenous opioid system is also involved in the modulation of social behaviour. As well as mediating physical pain [35, 36], the opioid system plays a key role in social attachment, affiliative behaviour and emotion regulation. Indeed, opioid receptors are abundantly expressed in brain regions central to the regulation of social and emotional behaviour [37–39]. Specifically, β-endorphin, which binds to µ-opioid receptors, modulates social motivation and is important for the formation and maintenance of social bonds [40–43]. Activation of the μ-opioid system is associated with increased sociability [44, 45], while the µ-opioid antagonist naltrexone diminishes feelings of social connection in people [46] and inhibits social behaviour in rodents [43, 45]. Notably, μ-opioid receptor knockout mice display symptoms characteristic of autism, with impairments in social interactions, communication and attachment behaviour [47–49]. Furthermore, the endogenous opioid system regulates both oxytocin and vasopressin release and there is considerable interaction between these three neuropeptide systems [49–53].

Neuropeptides are postulated to play an important role in communication between the gut microbiome and the brain, especially since they interact with both the immune system and the vagus nerve [54]. Interestingly, μ-opioid receptors are not only widely expressed in the brain but also in the gut, where they regulate the gut–brain neural circuitry involved in satiety [55]. In fact, opioids, oxytocin and vasopressin can all influence gut physiology, such as motility [56–59]. Certain *Lactobacillus* species can induce μ-opioid receptor expression in the gut via the nuclear factor-κB immune response [60], while antibiotic treatment has been found to reduce expression of this receptor in the gut [61]. However, the interaction between the gut microbiome and the μ-opioid system in the central nervous system has not previously been investigated. Furthermore, there have been few studies on the relationship between the gut microbiome and brain neuropeptide systems [13, 23], with research focusing on the expression of the peptides rather than their receptors.

Here we examine the influence of antibiotic treatment and germ-free status on neuropeptide pathways implicated in social and emotional behaviour by measuring gene expression of both the peptides and their corresponding receptors. The experiments were conducted in mice since they are a naturally social mammalian species and therefore represent a suitable model organism [62]. We adopt two different approaches, using mice treated with antibiotics post-weaning and germ-free mice, since both models are commonly used to ascertain the role of the microbiome in host development and behaviour.

## Methods

### Animals

For the antibiotic experiment, suckling male NIH Swiss mice (Charles River) at postnatal day 16 were housed with dams in cages maintained under standard controlled laboratory conditions with a 12 h light–dark cycle at 21 ± 1 °C and 50 ± 5% relative humidity. At postnatal day 21, mice were weaned and housed three per cage under identical environmental conditions and fed a standard mouse chow diet ad libitum. The animals were allocated randomly to the treatment and control groups and were provided with either drinking water containing a cocktail of antibiotics or drinking water alone, respectively. For the germ-free experiment, Swiss Webster mice were housed by the supplier (Taconic Biosciences) with three to five mice per cage in a gnotobiotic isolator under a 12 h light–dark cycle at 21 ± 1 °C and an average relative humidity of 50%. Mice were fed an autoclaved chow diet ad libitum and specific pathogen-free mice received chlorinated water, while germ-free mice received water that had been chlorinated and then autoclaved. The same strain, supplier and age (8 weeks) of germ-free and specific pathogen-free mice were used as in the seminal study showing reduced sociability in germ-free mice [12]. In both the antibiotic and germ-free experiments there were eight male animals in each of the treatment and control groups (*n* = 8, weight range 29–38 g at 8 weeks). This research was carried out with local ethical approval and a UK Home Office licence granted under the Animals (Scientific Procedures) Act 1986.

### Antibiotic treatment

This method followed that of a previous study which had shown that an antibiotic cocktail administered in early adolescence altered cognitive, social and emotional behaviour [23]. Mice were treated with antibiotics from postnatal day 21 to deplete the microbiota. This high-dose antibiotic cocktail administered in the drinking water comprised ampicillin (1 mg/ml), vancomycin (5 mg/ml), neomycin (10 mg/ml), metronidazole (10 mg/ml) and amphotericin-B (0.1 mg/ml). This specific mixture has been shown to reduce faecal bacterial DNA load by 400 fold without causing morbidity [63]. It is a commonly used broad-spectrum antibiotic treatment in gut microbiome research and is considered a standard microbiome depletion protocol [64–66]. Fluid intake was monitored every two days and the concentrations of antibiotics were adjusted according to the volume of fluid consumed. Mice were weighed routinely to monitor their general health and check that antibiotic treatment did not significantly affect body mass (Additional file 1: Figure S1). Animals were treated with antibiotics until postnatal day 55, when they were sacrificed.

### Tissue dissection

Following euthanasia via cervical dislocation, the brains of the antibiotic-treated and control mice were dissected into the frontal cortex, hippocampus, hypothalamus and brainstem and frozen at – 80 °C. The brain samples from germ-free and specific pathogen-free mice were placed in RNAlater (Sigma-Aldrich), transported on dry ice and then frozen at – 80 °C, prior to dissection into the same regions.

### RNA extraction and cDNA synthesis

The mass of each brain region was measured (to the nearest mg) prior to homogenizing the tissue in TRI Reagent (Sigma-Aldrich) for RNA extraction. The RNA concentration of each sample was measured using a NanoDrop ND-1000 spectrophotometer (Thermo Scientific) and each RNA extract was assessed for purity by ensuring the 260/280 nm absorbance ratio was approximately two. Each RNA sample was diluted in nuclease-free water (Millipore) and stored at – 20 °C. In preparation for reverse transcription, the RNA was treated with DNase (Promega) and heated to 37 °C for 20 min, followed by 5 min at 75 °C. Complementary DNA (cDNA) was synthesized from 1 µg RNA using the High-Capacity cDNA Reverse Transcription Kit (Applied Biosystems) and incubated at 37 °C for 1 h, along with a corresponding negative control for each sample. The cDNA samples were then diluted in nuclease-free water to a concentration of 20 ng/µl, prior to storage at – 20 °C.

### Quantitative real-time PCR

Quantitative real-time polymerase chain reaction (RT-PCR) was performed on the 7900HT Fast Real-Time PCR System (Applied Biosystems), with all cDNA samples diluted in nuclease-free water to a concentration of 5 ng/µl. Each RT-PCR reaction consisted of 3 µl cDNA, 6 µl TaqMan Universal PCR Master Mix (no AmpErase UNG) and 0.6 µl TaqMan probes, and was made up to a total volume of 12 µl with nuclease-free water. The same thermal cycling conditions were used for all runs: 5 min at 95 °C (initial step), 15 s at 95 °C (denaturation step) and 45 s at 60 °C (annealing and extension step), with the latter two steps repeated for 40 cycles. TaqMan gene expression assays (Additional file 1: Table S1) were used to quantify messenger RNA (mRNA) expression of genes encoding the µ-opioid (*Oprm1*), oxytocin (*Oxtr*) and vasopressin (*Avpr1a*) receptors and their corresponding peptides, namely pro-opiomelanocortin (*Pomc*), oxytocin (*Oxt*) and vasopressin (*Avp*) respectively (note that pro-opiomelanocortin is cleaved into multiple peptides, including β-endorphin which binds to µ-opioid receptors). While there are several opioid and vasopressin receptor genes, we measured the expression of *Oprm1* and *Avpr1a* since these are most strongly associated with social behaviour [43, 45, 67, 68].

In addition, gene expression levels were also determined for myeloid differentiation factor 88 (*MyD88*) which is a key mediator of host–microorganism communication and integral to the host immune response and intestinal homeostasis [64, 69]. Expression of the gene encoding brain-derived neurotrophic growth factor (*Bdnf*), important for neuronal growth and survival, was measured as a positive control since several studies have reported its downregulation in both antibiotic-treated mice [23, 24, 28] and germ-free mice [70–72]. Negative controls (both no-template and nuclease-free water) were also included in each run for the treatment and control groups. All samples were run in triplicate and the average threshold cycle number (C_T_) recorded. As recommended for accurate normalization of RT-PCR data [73], C_T_ values for the target genes were normalized to the geometric mean of three endogenous control genes (*Gapdh*, *B2m* and *Polr2a*) whose expression did not differ significantly between the control and treatment groups.

### Statistical analyses

Data were analysed using the comparative C_T_ method [74, 75] and two-sample t-tests were performed on the $2^{- \Delta \Delta{\text{C}_{\text{T}}}}$ values to test for statistically significant differences in gene expression between the control and treatment groups. Where necessary, Welch’s t-test was conducted when the variances of the two groups were significantly different (determined using Levene’s test for homogeneity of variances) and the non-parametric Mann–Whitney U test was carried out when the assumption of normality was not met (determined using the Shapiro–Wilk test).

## Results

### Antibiotic-treated mice

**Fig. 1:**
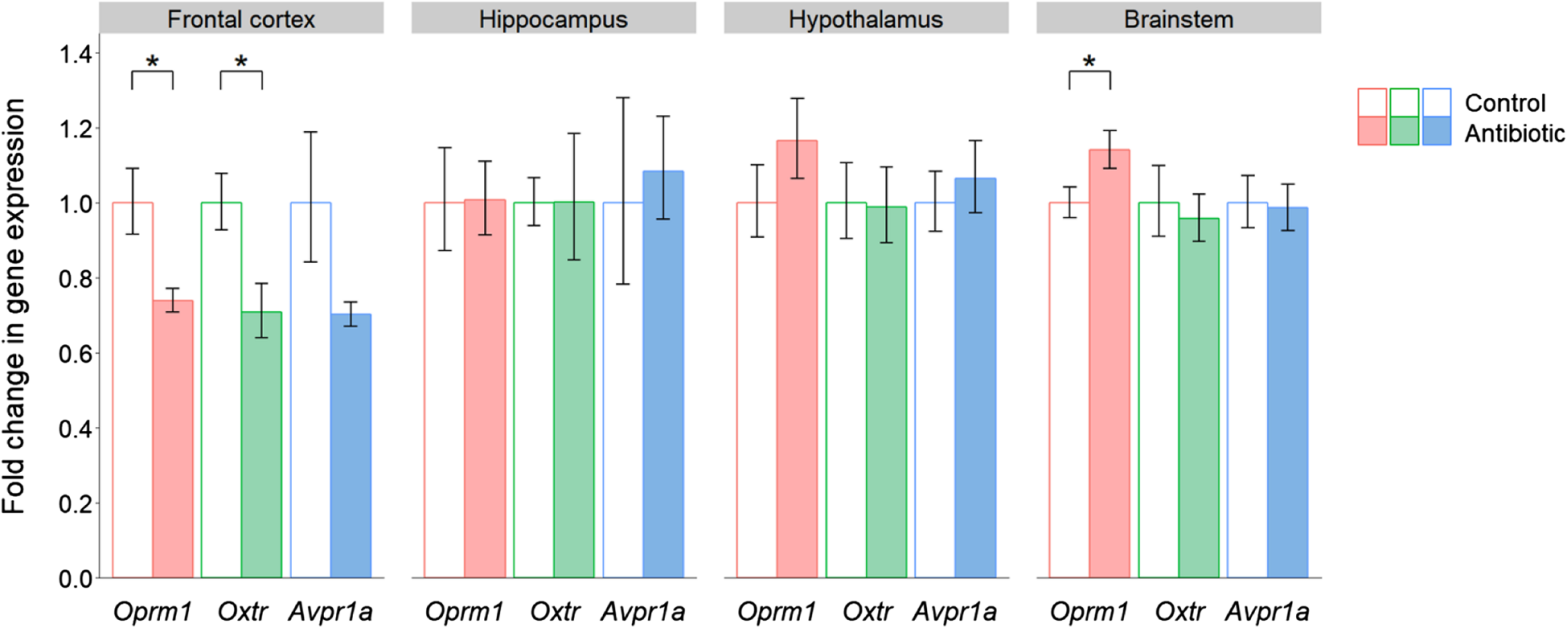
Effects of post-weaning antibiotic treatment on the expression of neuropeptide receptor genes implicated in social behaviour. Data are plotted as mean expression (relative to the control group) ± SEM for *Oprm1*, *Oxtr* and *Avpr1a* (encoding the µ-opioid, oxytocin and vasopressin receptors respectively) and all comparisons are with *n* = 8 per group. Two-sample t-tests have 14 degrees of freedom (df) and asterisks denote *P* < 0.05

The most striking effect of antibiotic administration was in the frontal cortex, where all three receptor genes showed a reduction in expression (Fig. 1). This downregulation in the treatment group was significant for both *Oprm1* (*P* = 0.021) and *Oxtr* (*P* = 0.016), with a trend towards reduced expression for *Avpr1a* (*P* = 0.079). While there were no significant differences in receptor gene expression in the hippocampus or hypothalamus, there was a significant increase in *Oprm1* expression in the brainstem (*P* = 0.046).

**Fig. 2:**
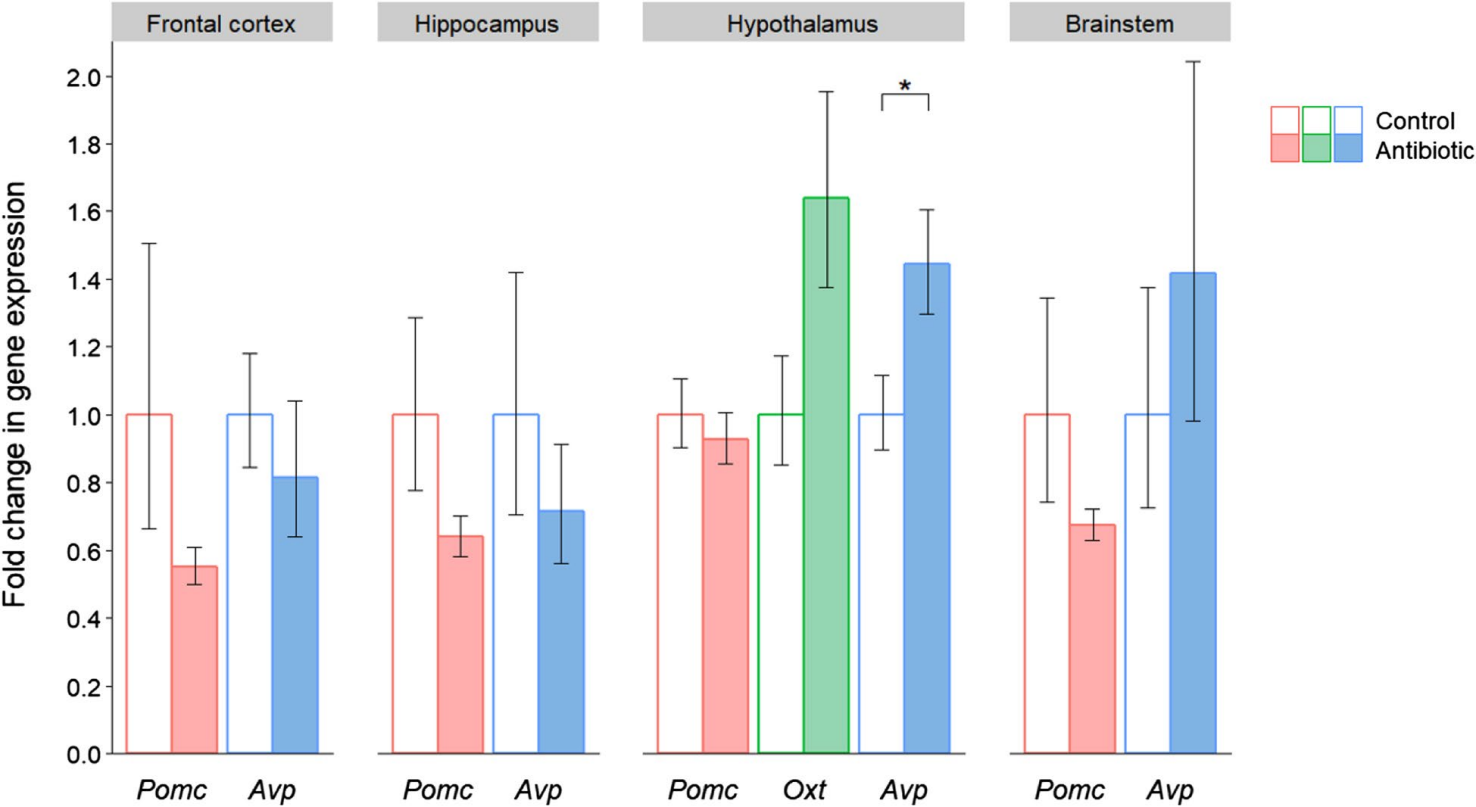
Effects of post-weaning antibiotic treatment on the gene expression of neuropeptides implicated in social behaviour. Data are plotted as mean expression (relative to the control group) ± SEM for *Pomc*, *Oxt* and *Avp* (encoding the peptides pro-opiomelanocortin, oxytocin and vasopressin respectively) and all comparisons are with *n* = 8 per group. Two-sample t-tests have 14 degrees of freedom (df) and asterisks denote *P* < 0.05. Note that *Oxt* was only detectable in the hypothalamus, its site of production

In terms of the corresponding peptides (Fig. 2), the expression of *Pomc* and *Avp* was reduced in the frontal cortex and hippocampus of the antibiotic-treated mice. However, these differences were not significant due to the considerable within-group variation in peptide gene expression. In the hypothalamus there was increased expression of both *Oxt* (*P* = 0.065) and *Avp* (*P* = 0.037), which was statistically significant in the case of *Avp*. In addition, there was a consistent trend towards reduced *Pomc* expression in each of the four brain regions, and when the results from all regions were combined, this downregulation was statistically significant (*P* = 0.010).

**Fig. 3:**
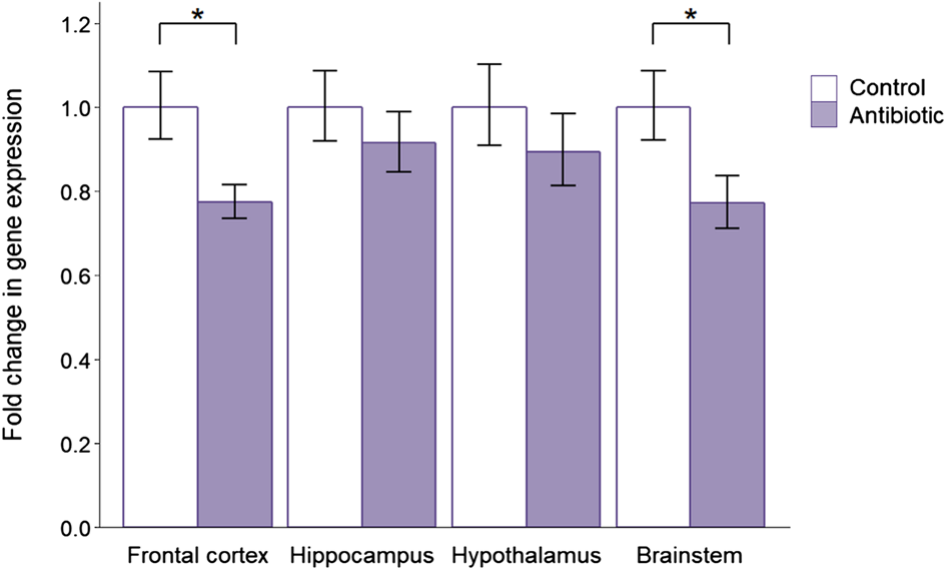
Effects of post-weaning antibiotic treatment on the gene expression of *MyD88*. Data are plotted as mean expression (relative to the control group) ± SEM for *MyD88* (encoding myeloid differentiation factor 88) and all comparisons are with *n* = 8 per group. Two-sample t-tests have 14 degrees of freedom (df) and asterisks denote *P* < 0.05

*MyD88* expression was reduced in all four brain regions of antibiotic-treated mice (Fig. 3), with a significant downregulation in the frontal cortex (*P* = 0.021) and brainstem (*P* = 0.043). Antibiotic administration also led to reduced expression of *Bdnf* (included as the positive control gene) in the frontal cortex (*P* = 0.013, Additional file 1: Figure S2). This is in accordance with the majority of previous research showing a decrease in *Bdnf* expression in antibiotic-treated mice [23, 24, 28], thereby validating the treatment effects.

### Germ-free mice

**Fig. 4:**
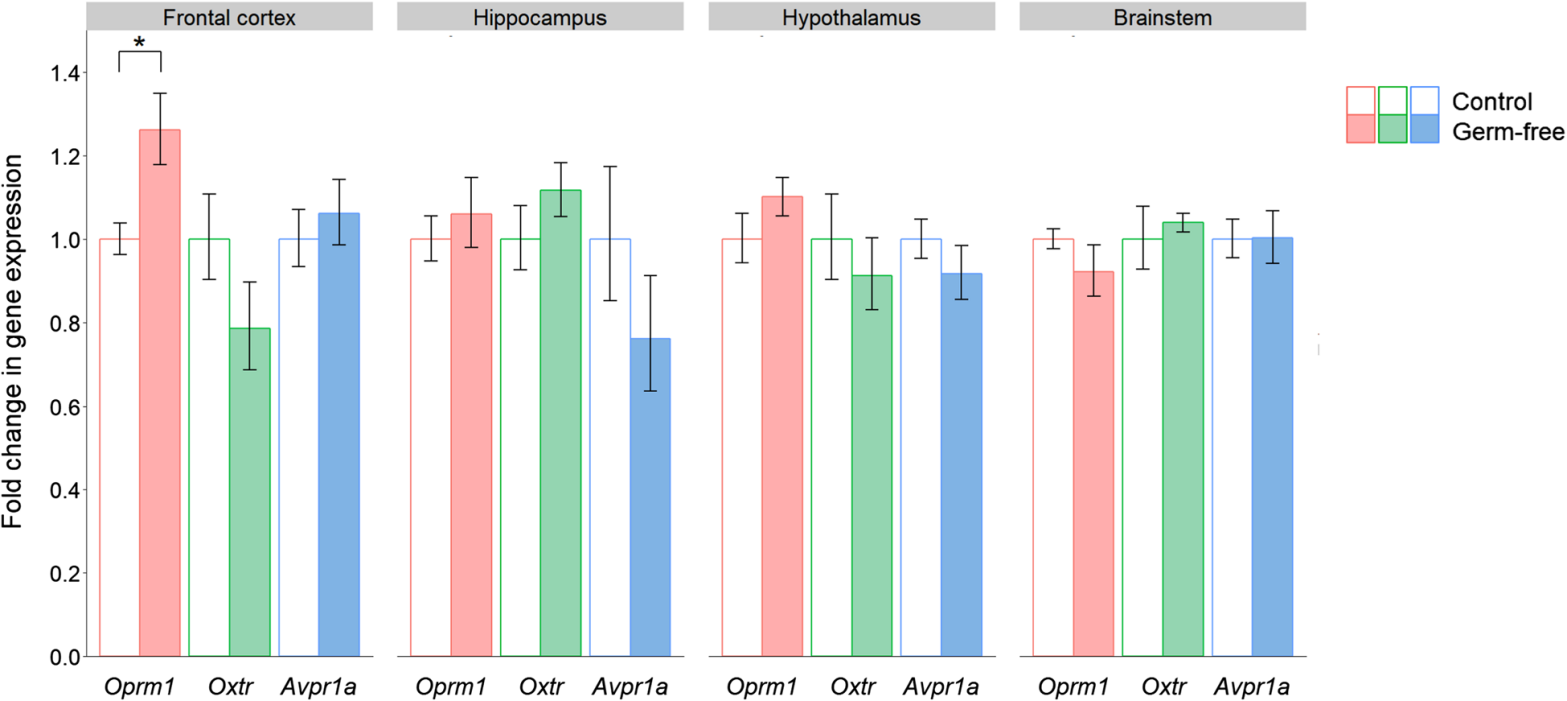
Effects of germ-free status on the expression of neuropeptide receptor genes implicated in social behaviour. Data are plotted as mean expression (relative to the control group) ± SEM for *Oprm1*, *Oxtr* and *Avpr1a* (encoding the µ-opioid, oxytocin and vasopressin receptors respectively) and all comparisons are with n = 8 per group. Two-sample t-tests have 14 degrees of freedom (df) and asterisks denote P < 0.05

The only significant change in receptor gene expression was for *Oprm1* in the frontal cortex (Fig. 4). Notably the treatment effect was in the opposite direction to antibiotic-treated mice since *Oprm1* expression was upregulated in the frontal cortex of germ-free mice (*P* = 0.018).

**Fig. 5:**
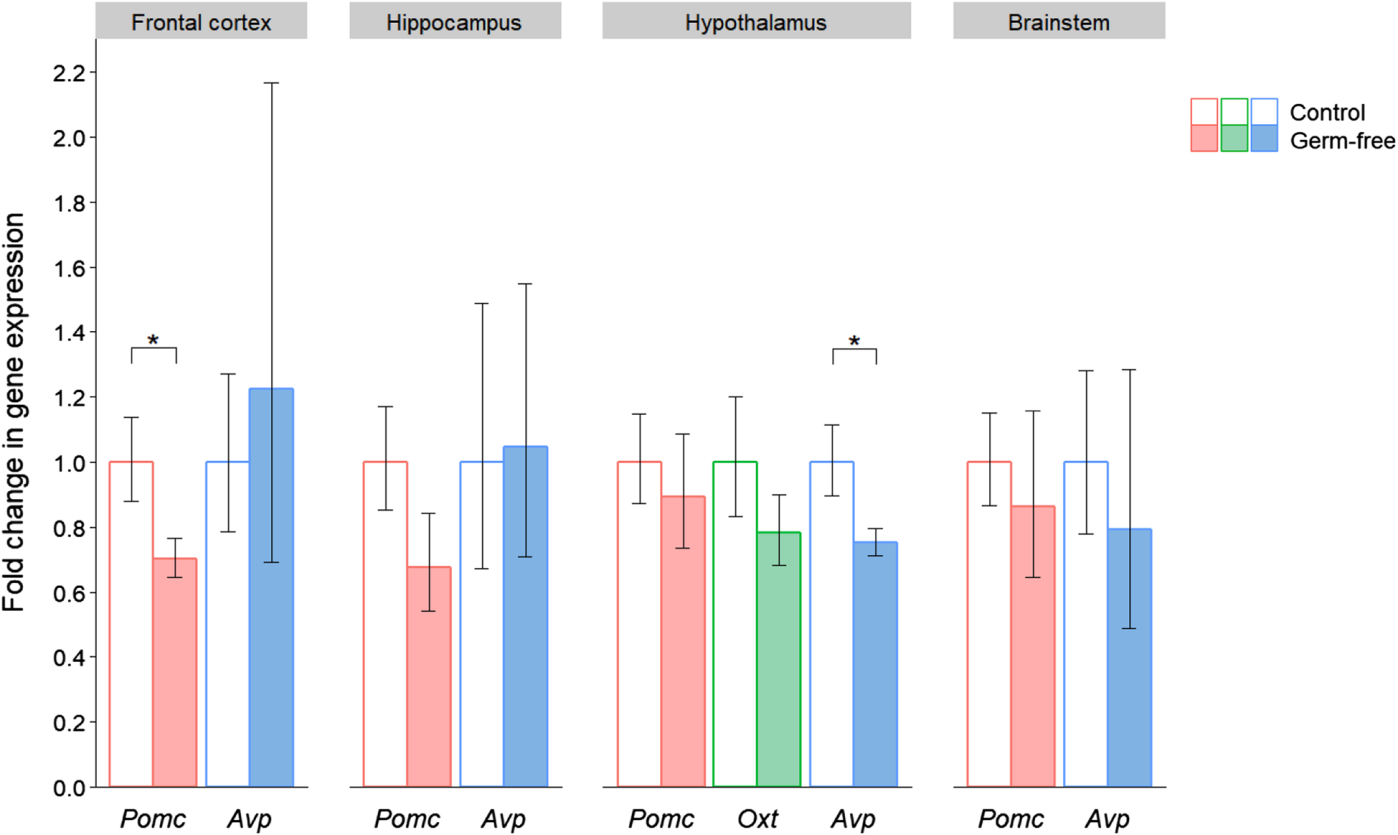
Effects of germ-free status on the gene expression of neuropeptides implicated in social behaviour. Data are plotted as mean expression (relative to the control group) ± SEM for *Pomc*, *Oxt* and *Avp* (encoding the peptides pro-opiomelanocortin, oxytocin and vasopressin respectively) and all comparisons are with *n* = 8 per group. Two-sample t-tests have 14 degrees of freedom (df) and asterisks denote *P* < 0.05

Regarding peptide gene expression (Fig. 5), *Avp* was downregulated in the hypothalamus of germ-free mice (*P* = 0.046), which again contrasted the direction of change in antibiotic-treated mice. *Pomc* expression was significantly reduced in the frontal cortex (*P* = 0.030) and combining the expression results from all four brain regions also revealed a significant overall downregulation of *Pomc* (*P* = 0.008), as was the case in the antibiotic-treated animals.

**Fig. 6:**
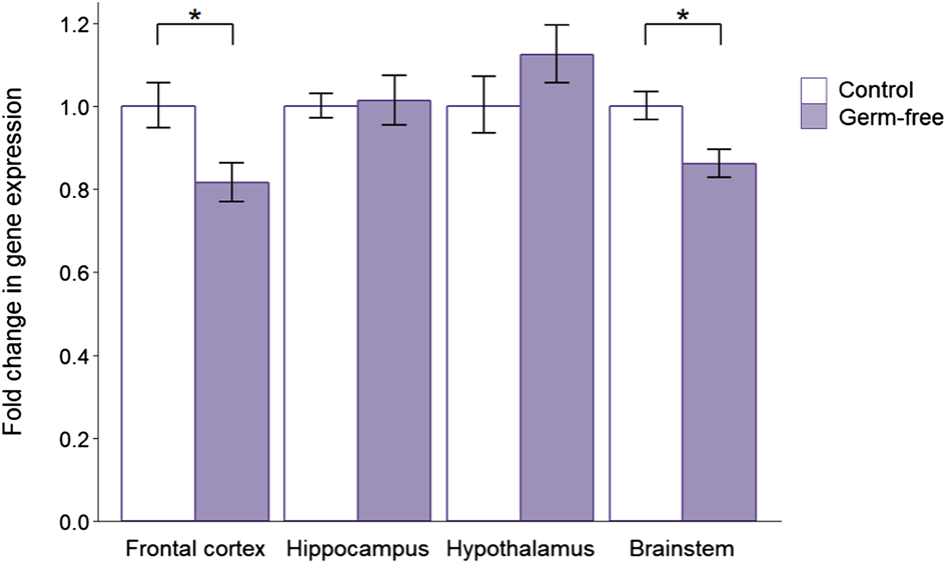
Effects of germ-free status on the gene expression of *MyD88*. Data are plotted as mean expression (relative to the control group) ± SEM for *MyD88* (encoding myeloid differentiation factor 88) and all comparisons are with *n *= 8 per group. Two-sample t-tests have 14 degrees of freedom (df) and asterisks denote *P* < 0.05

*MyD88* expression was significantly reduced in the frontal cortex (*P* = 0.019) and brainstem (*P* = 0.012) of germ-free mice (Fig. 6), as in the antibiotic treatment group. Similarly, *Bdnf* expression in germ-free mice (Additional file 1: Figure S3) was also reduced in the frontal cortex (*P* = 0.050) and brainstem (*P* = 0.005). These changes in the positive control gene are consistent with the majority of previous findings showing downregulation of *Bdnf* in germ-free mice [23, 70–72].

## Discussion

In this study we show that the gene expression of social neuropeptides and their receptors is altered in both antibiotic-treated and germ-free mice, as summarised in Table 1. The most notable finding is the reduction in neuropeptide receptor gene expression in the frontal cortex of mice administered the antibiotic cocktail, which is particularly relevant given the prominent role of this brain region in social behaviour [76]. Downregulation of these receptors may partly reflect the considerable interaction between the μ-opioid, oxytocin and vasopressin systems [49–53]. The behavioural impairments reported in antibiotic-treated rodents [19, 22, 23, 25–29], including mice receiving the identical combination of antibiotics used in this study [23], may therefore partially be a result of reduced activation of these neuropeptide pathways.

**Table 1:**
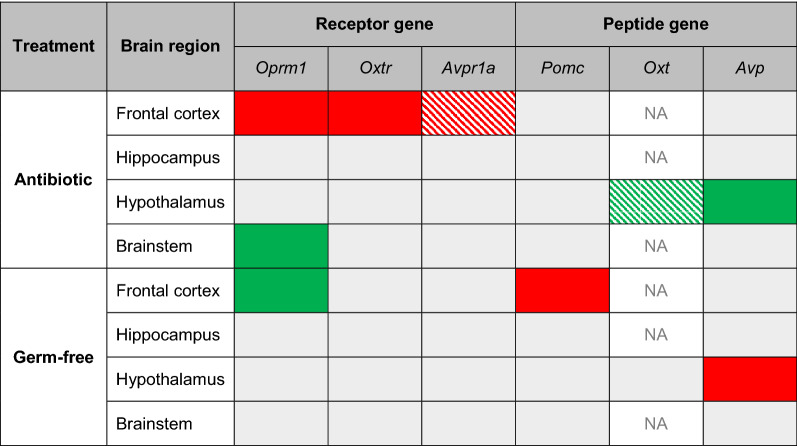
Summary of results for changes in expression of neuroreceptor and neuropeptide genes in the antibiotic-treated and germ-free mice

Cells coloured red denote a significant reduction in gene expression and those coloured green denote a significant increase in gene expression. Hatch patterns indicate non-significant trends. Note that *Oxt* was only detectable in the hypothalamus, its site of production

In contrast to the downregulation of *Oprm1* (encoding the μ-opioid receptor) in the frontal cortex of antibiotic-treated mice, this gene was significantly upregulated in the brainstem. Notably, research on the serotonergic system has also reported elevated signalling in the brainstem and reduced serotonin turnover in the frontal cortex of suicides with a psychiatric diagnosis [77]. This similar pattern of regional differences in the μ-opioid system may therefore reflect the known interaction between serotonin and opioid signalling [78–80]. The antibiotic-treated mice also showed an increase in expression of *Avp* (encoding vasopressin) and a trend towards upregulation of *Oxt* (encoding oxytocin) in the hypothalamus, their site of synthesis, perhaps compensating for the corresponding reduction in receptor gene expression in the frontal cortex. Interestingly, a similar pattern of increased gene expression of the peptide but downregulation of its receptor was also reported for the neuropeptide Y pathway in antibiotic-treated mice [24]. The upregulation of *Avp* found here is in the opposite direction to a previous study using the same antibiotic treatment and mouse strain, where *Avp* expression was reduced in the hypothalamus [23]. One important difference, however, is that our animals were behaviourally naïve rather than having been subjected to a series of behavioural tests prior to measuring gene expression. In contrast to the increased expression of *Oxt* and *Avp* in our antibiotic treatment group, *Pomc* showed an overall reduction in expression when the results of all four brain regions were combined. This suggests reduced endorphin levels in the brains of antibiotic-treated mice, since the peptide encoded by *Pomc* is cleaved to produce β-endorphin. Combined with reduced expression of the corresponding receptor gene *Oprm1*, these data indicate that μ-opioid signalling may be attenuated in these animals. In fact, reduced activation of the μ-opioid system has been associated with social deficits [43, 45], and such deficits are typically observed in rodents administered antibiotics [23, 27–29].

The one previous study looking at the effect of antibiotic treatment on the neuropeptides oxytocin and vasopressin only measured gene expression of the peptide and not its corresponding receptor [23]. Our results indicate that quantifying mRNA abundance for the receptors, rather than the neuropeptides, may be more informative since they showed considerably less variation in gene expression. Perhaps this is because receptor transcript levels are more stable, with a lower turnover than signalling peptides. Thus, measuring both peptide and receptor gene expression may allow a more holistic assessment of the disruption of brain signalling pathways and the possible compensatory mechanisms involved.

It might be expected that germ-free conditions would have similar effects on gene expression as antibiotic exposure, and perhaps more extreme given the complete lack of microorganisms, rather than just a severe depletion. Surprisingly, the significant changes in expression were generally in the opposite direction to the antibiotic-treated mice. *Avp* was downregulated in the hypothalamus of germ-free mice but upregulated in the hypothalamus of antibiotic-treated mice. *Oprm1* was upregulated in the frontal cortex of germ-free mice, in contrast to its decreased expression in antibiotic-treated mice. Interestingly, germ-free mice showed reduced *Pomc* expression in the frontal cortex and this pattern of peptide downregulation and receptor upregulation in germ-free mice may reflect a homeostatic mechanism. In fact, there is evidence of feedback inhibition in the opioid system whereby high levels of receptor activation inhibit pro-opiomelanocortin neurone activity, resulting in lower concentrations of the peptide [81].

In light of the known interactions between these neuropeptides and the immune system [82–84] and the increasing evidence that the immune system is an important mediator of the microbiome–gut–brain axis [85], we were also interested to investigate the effect of both antibiotic treatment and germ-free status on *MyD88* expression. This gene encodes an immune system protein which is a key mediator of host–microorganism communication in vertebrates. Specifically, MyD88 functions as an adaptor protein for Toll-like receptors, which detect and respond to gut bacteria in the intestinal epithelium [64, 69, 86]. MyD88 signalling therefore plays an integral role in the host’s immune response [69, 87–89] and is also important for homeostasis of the intestinal barrier [64, 90, 91]. Both antibiotic treatment and germ-free status had the same effect on *MyD88* expression, with a significant reduction in both the frontal cortex and brainstem. Since commensal microbiota drive stimulation of MyD88, its downregulation may reflect the reduction in microbial interactions with Toll-like receptors due to the lack of microorganisms in antibiotic-treated and germ-free mice. In fact, there is evidence that both probiotics and prebiotics benefit gut health by stimulating the immune system through MyD88-dependent NF-κB activation [92–96]. Moreover, *MyD88* is expressed in many different organs and cell types beyond the gut, including neurones [97] and microglia [98], suggesting that it may also be involved in communicating the state of the gut to distant organs such as the brain. Future studies using immunodeficient animals, such as *MyD88* conditional knockout mice, will be required to experimentally determine its role in the microbiome–gut–brain axis.

Overall, our findings that neuropeptide pathways implicated in social and emotional behaviour are disrupted are in line with previous studies showing social impairments in antibiotic-treated and germ-free rodents [11–13, 15, 16, 23, 27–29]. However, while these animal models may be useful for establishing causality, we cannot necessarily extrapolate from these findings the role of the gut microbiome in the social behaviour of healthy animals. Future studies involving more fine-scale manipulations of the gut microbial community, such as probiotic or prebiotic feeding or narrow-spectrum antibiotic administration, are required to determine the contribution of specific members of the gut microbiota. While the use of antibiotics to deplete the gut microbiota is often seen as a more accessible alternative to germ-free models [23, 63, 65], the differences reported here between antibiotic-treated and germ-free mice suggest that they should be viewed as distinct models of gut microbiome manipulation. Indeed, antibiotic-treated mice have a normal gut microbiome during the early postnatal period whereas germ-free mice represent a rather different biological case since these animals are never exposed to microorganisms and so exhibit impaired physiology and immune development from birth. However, it cannot be eliminated that differences between the antibiotic-treated and germ-free mice may be partly due to the different mouse strains, though both were outbred strains. The antibiotic experiment was conducted with NIH Swiss mice since this was the same strain used by a previous study which had demonstrated effects of this antibiotic treatment on the brain and behaviour [23], and whose methodology was closely followed here. The germ-free experiment was carried out with Swiss Webster mice since this is the strain most commonly available as a germ-free model and is the primary strain in studies reporting impaired sociability in germ-free mice [12, 14, 15, 18].

To date, there have been few studies investigating the effects of antibiotics on the brain during early life [99], yet antibiotic treatment is arguably more clinically relevant than germ-free conditions. In fact, antibiotics are the most commonly prescribed drug to children [100] and human population studies have linked antibiotic exposure during early life with increased risk of allergy, inflammatory bowel disease and obesity, as well as poorer neurocognitive outcomes [101]. The results here, particularly the salient reduction in neuroreceptor gene expression in the frontal cortex, have potential implications for understanding the impact of antibiotic administration on human brain function. Furthermore, the interaction between the gut microbiome and the brain’s endogenous opioid system has not previously been investigated, with the results of this study indicating reduced μ-opioid signalling in mice treated with antibiotics in early adolescence. This may at least partly explain the behavioural deficits in animals exposed to antibiotics [23, 27–29] since activation of the μ-opioid system is integral to social motivation and social bonding [43, 45], as well as regulating stress and emotion [39, 102, 103]. While caution must be exercised when considering the possible impact of antibiotics during childhood and adolescent years in humans, we hypothesize that the μ-opioid system may also be downregulated, with implications for behaviour. Notably, μ-opioid receptor density in the human frontal cortex is positively associated with motivation to seek close social relationships [104], which is especially relevant given the reduced gene expression of this receptor in the frontal cortex of antibiotic-treated mice. Human brain imaging studies have also revealed that activation of the μ-opioid system helps to alleviate the experience of social pain and is positively correlated with social motivation and psychological resilience [105, 106]. Thus a decrease in μ-opioid signalling may have consequences for mental health and well-being. In fact, dysregulation of the endogenous opioid system has been implicated in depression and schizophrenia, with the majority of human studies reporting a reduction in β-endorphin levels [107, 108] or decreased activity of the μ-opioid system [37, 106]. It is an interesting observation that antibiotic exposure in humans is associated with increased risk of depression and anxiety [109] and treatment with antibiotics during the first year of life has also been linked to an increased risk of depression and behavioural difficulties in childhood [110]. However, further research is required to determine the changes in brain function that may underlie these associations and whether the effects are long-lasting.

As well as its role in social behaviour, the endogenous opioid system is a key regulator of pain [35, 36]. Our finding that antibiotic treatment reduces μ-opioid receptor expression in the central nervous system therefore has potential clinical relevance. In fact, antibiotic treatment in rodents increases visceral pain [19, 21], including early-life antibiotic exposure [22]. Whilst the gut microbiome has been shown to modulate pain, the mechanisms are yet to be elucidated [111], though our results suggest that the effect of the microbiome on both pain and social behaviour may be underpinned, at least in part, by its interaction with the μ-opioid system. In support of this, a recent study showed that decreased expression of *Oprm1* in the prefrontal cortex was associated with increased pain sensitivity in male and female rats [112]. A reduction in μ-opioid receptor availability in the brain has been linked to increased pain sensitivity in healthy volunteers [113] and has also been found in sufferers of chronic pain [114]. In addition, since the opioid system is central to addiction [115] and we show that the gut microbiome interacts with this neuropeptide system, our findings are also relevant to the observation that antibiotic treatment enhances addictive behaviour in mice [116].

In terms of further research directions, it would be interesting to investigate whether prebiotic or probiotic supplementation in antibiotic-treated animals can rescue the disrupted social neuropeptide signalling, and whether any effects of probiotics are specific to certain bacterial species or strains. Since antibiotic treatment can deplete the production of short-chain fatty acids, particularly butyrate [117, 118], it would also be informative to test whether administration of butyrate helps to reduce the impact of antibiotic treatment. If so, this would suggest that the influence of the gut microbiome on the expression of these neuropeptide pathways is at least partly mediated by butyrate. Future research should also examine the impact of antibiotic administration on the neurogenetics of adult mice to determine whether the effect is less marked than antibiotic treatment immediately following weaning when the developing brain is particularly vulnerable to perturbation. To avoid introducing additional variation, this study was only conducted on male mice but it would be interesting to investigate whether females show similar changes in gene expression since there are some notable sex differences in the microbiome–gut–brain axis [27, 119–121]. Indeed, a recently reported study found that early-life antibiotic exposure induced social deficits only in male mice [29]. Similarly, male germ-free mice show more pronounced social impairments [12] and alterations in neurochemistry [72, 122] compared to their female counterparts, which may be reflective of the higher incidence of autism among males. It should also be noted that this study focused on quantifying mRNA expression. This is considered to provide a more accurate indication of treatment-induced changes in neuronal gene expression than measuring protein levels which may reflect homeostatic balance between neuronal release, translation and storage [24]. While there is a degree of correspondence between mRNA and protein levels [123, 124], protein abundance cannot necessarily be inferred from quantifying mRNA transcripts. However, studies on these neuropeptide systems in rodents suggest that changes in mRNA expression have measurable impacts on behaviour and pain response [125–127].

While antibiotic treatment and germ-free conditions are two helpful approaches to investigate causality, both models have limitations. Germ-free animals show numerous defects, including in their development, gut morphology and physiology, nervous system, immune response and metabolism [128–131]. Notably, they also have a more permeable blood–brain barrier [132]. Although this artificial model is useful for investigating which processes are modulated by the gut microbiome, the findings are not easily translatable to human physiology and disease. In terms of the high-dose, broad-spectrum antibiotic treatment used in this study, it not only dramatically reduces the bacterial load but also significantly restructures the gut microbial community, decreasing its diversity and altering its composition [23, 118], including the possibility of increasing antibiotic-resistant bacteria. While the relative abundances of the phyla Firmicutes and Bacteroidetes are reduced, Cyanobacteria and Proteobacteria increase in relative abundance with this antibiotic cocktail [23, 118]. Indeed, the phylum Proteobacteria includes a wide variety of pathogens such as *Escherichia*, *Helicobacter*, *Pseudomonas*, *Salmonella* and *Vibrio* [133] and increased abundance of this phylum is associated with psychological distress in patients with irritable bowel syndrome [134]. In addition, members of the phylum Cyanobacteria produce toxins including lipopolysaccharides which can have pathological effects [135]. Thus, the effects of antibiotic treatment may not necessarily be through depleting the gut microbiota but also by significantly changing its composition. Furthermore, antibiotics can have systemic effects in addition to their antimicrobial actions [130, 136]. Antibiotics may even act directly on the brain and there is evidence that they can modulate the vagus nerve [137] and the enteric nervous system [138]. Antibiotics can also have neurotoxic effects including encephalopathy [139], as observed in some clinical cases of metronidazole use [140]. Thus while this is a widely used antibiotic cocktail to deplete the microbiota [64–66], including in studies of the microbiome–gut–brain axis [19, 23], it is important to bear in mind that observed changes may be partly the result of systemic effects of antibiotics, in addition to the direct effects of antibiotics on reducing bacterial load. Finally, although mice represent a useful model for understanding mammalian physiology, with many similarities between the mouse and human gut microbiome, there are also notable differences and caution is needed when extrapolating the potential significance of these findings to humans [130, 141].

## Conclusions

The results of our study reveal that disruption of the gut microbiome in early life can significantly impact social neuropeptide signalling. The reduced activation of these pathways in mice treated with antibiotics post-weaning likely contributes to their social impairments. It is also relevant to note the opposing effects of antibiotic treatment and germ-free status on gene expression in the brain; an interesting observation that should be explored in further studies. Our findings indicate the possible effects that early-life exposure to antibiotics may have on pathways in the brain mediating social and emotional behaviour, with potential implications for the risk of developing neuropsychiatric conditions such as autism, depression and anxiety.

## Supplementary information

**Additional file 1: Table S1.** Gene expression assays used for quantitative real-time PCR. **Fig. S1.** Effect of antibiotic treatment on body weight. **Fig. S2.** Effects of post-weaning antibiotic treatment on the gene expression of *Bdnf*. **Fig. S3.** Effects of germ-free status on the gene expression of *Bdnf*.

## Data Availability

Data are available from the author upon reasonable request.

## Abbreviations

*Avp*: Vasopressin gene
*Avpr1a*: Arginine vasopressin receptor 1A gene
*B2m*: Beta-2-microglobulin gene
*Bdnf*: Brain-derived neurotrophic factor gene
cDNA: Complementary DNA
C_T_: Threshold cycle number
df: Degrees of freedom
DNA: Deoxyribonucleic acid
*Gapdh*: Glyceraldehyde-3-phosphate dehydrogenase gene
mRNA: Messenger RNA
*MyD88*: Myeloid differentiation primary response 88 gene
*Oprm1*: Opioid receptor μ1 gene
*Oxt*: Oxytocin gene
*Oxtr*: Oxytocin receptor gene
PCR: polymerase chain reaction
*Polr2a*: RNA polymerase II subunit A gene
*Pomc*: Pro-opiomelanocortin gene
RNA: Ribonucleic acid
RT-PCR: Real-time PCR
SEM: Standard error of the mean
