# Supplementary material for: Opposing effects of antibiotics and germ-free status on neuropeptide systems involved in social behaviour and pain regulation
Source: BMC Neurosci. 2020 Jul 22;21:32. doi: 10.1186/s12868-020-00583-3 (PMC7374917; doi:10.1186/s12868-020-00583-3)
Supplement: Supplementary file 1 — Additional file 1: Table S1. Gene expression assays used for quantitative real-time PCR. Fig. S1. Effect of antibiotic treatment on body weight. Fig. S2. Effects of post-weaning antibiotic treatment on the gene expression of Bdnf. Fig. S3. Effects of germ-free status on the gene expression of Bdnf. [file 12868_2020_583_MOESM1_ESM.pdf]

Supplementary Information

Table S1 Gene expression assays used for quantitative real-time PCR

Gene	Protein encoded by gene	TaqMan gene expression assay ID
Oprm1	Opioid receptor µ1	Mm01188089_m1
Oxtr	Oxytocin receptor	Mm01182684_m1
Avpr1a	Arginine vasopressin receptor 1A	Mm00444092_m1
Pomc	Pro-opiomelanocortin	Mm00435874_m1
Oxt	Oxytocin	Mm01329577_g1
Avp	Vasopressin	Mm00437761_g1
MyD88	Myeloid differentiation factor 88	Mm00440338_m1
Bdnf	Brain-derived neurotrophic factor	Mm00432069_m1
Gapdh	Glyceraldehyde-3-phosphate dehydrogenase	Mm99999915_g1
B2m	Beta-2-microglobulin	Mm00437762_m1
Polr2a	Polymerase (RNA) II subunit A	Mm00839502_m1

All IDs refer to TaqMan assays (Applied Biosystems)

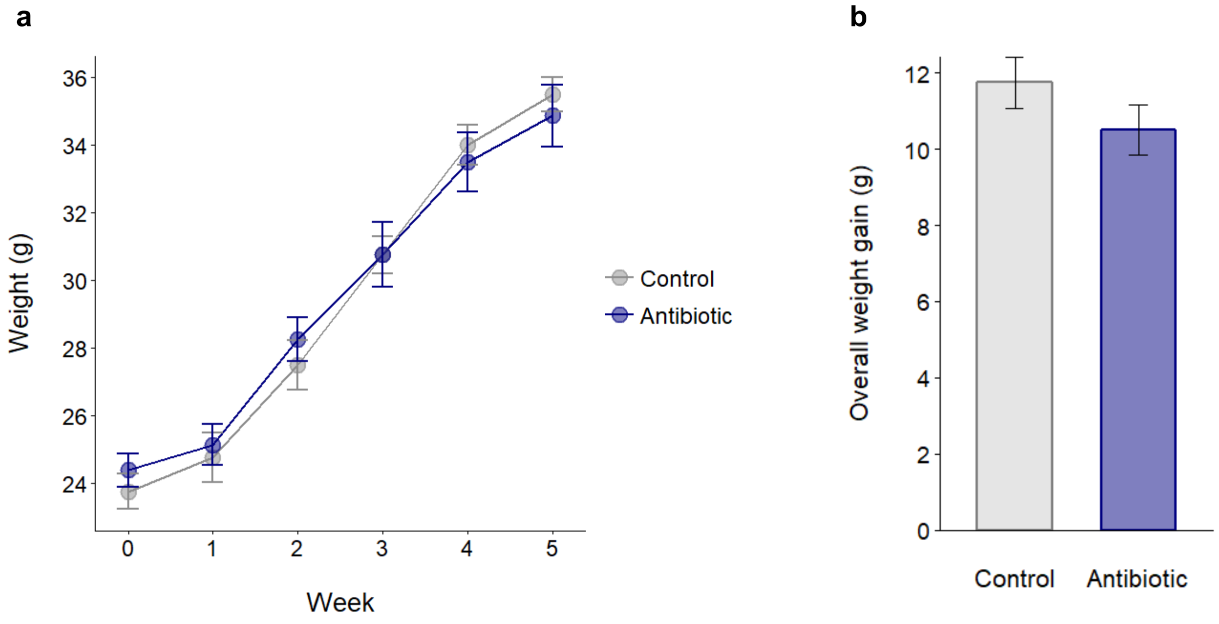

Fig. S1 Effect of antibiotic treatment on body weight. Data are plotted as mean weight change ± SEM, with $n = 8$ per group. (a) Line graph depicts weekly changes in weight for each group throughout the experiment. (b) Bar plot shows overall weight gain from prior to the start of antibiotic treatment (week 0) to the end of treatment (week 5). There was no significant difference in weight gain between groups (Mann–Whitney U test, $P = 0.284$)

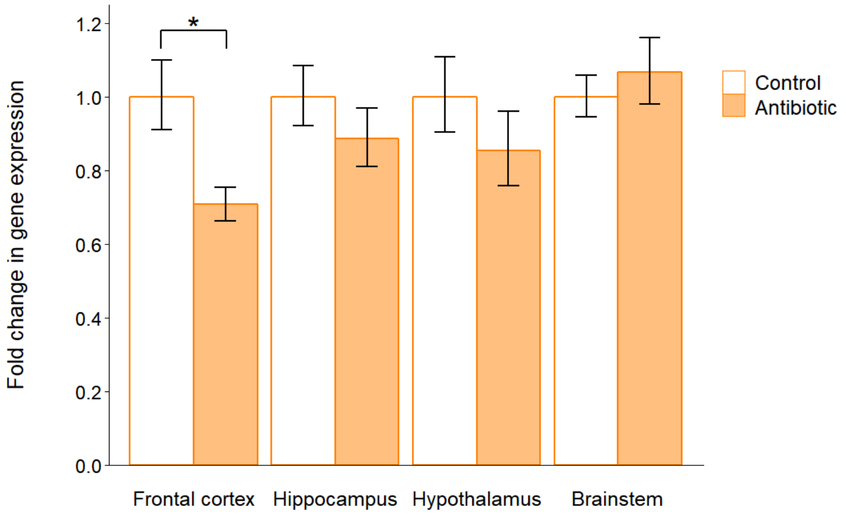

Fig. S2 Effects of post-weaning antibiotic treatment on the gene expression of *Bdnf*. Data are plotted as mean expression (relative to the control group) ± SEM for *Bdnf* (encoding brain-derived neurotrophic growth factor) and all comparisons are with n = 8 per group. Two-sample t-tests have 14 degrees of freedom (df) and asterisks denote $P < 0.05$

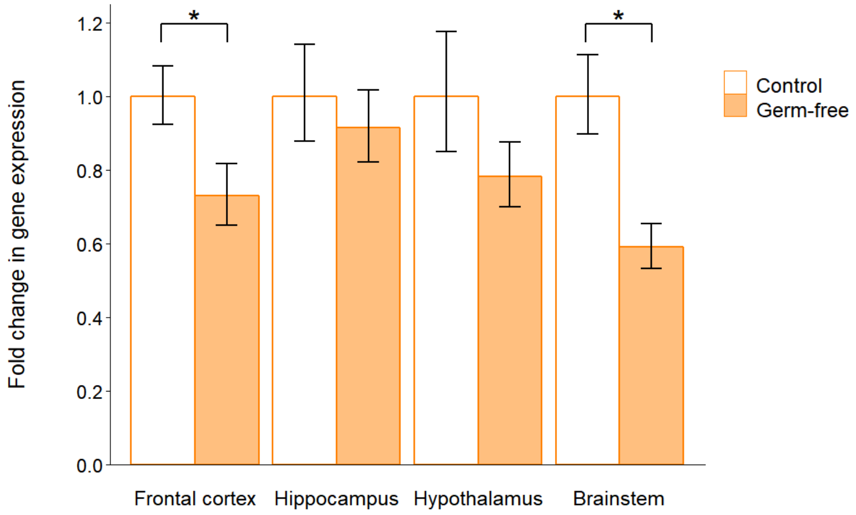

Fig. S3 Effects of germ-free status on the gene expression of *Bdnf*. Data are plotted as mean expression (relative to the control group) ± SEM for *Bdnf* (encoding brain-derived neurotrophic growth factor) and all comparisons are with n = 8 per group. Two-sample t-tests have 14 degrees of freedom (df) and asterisks denote $P < 0.05$
